# Arabidopsis flower specific defense gene expression patterns affect resistance to pathogens

**DOI:** 10.3389/fpls.2015.00079

**Published:** 2015-02-20

**Authors:** Luisa Ederli, Adam Dawe, Stefania Pasqualini, Mara Quaglia, Liming Xiong, Chris Gehring

**Affiliations:** ^1^Department of Chemistry, Biology and Biotechnology, University of PerugiaPerugia, Italy; ^2^Division of Biological and Environmental Sciences and Engineering, King Abdullah University of Science and TechnologyThuwal, Saudi Arabia; ^3^Department of Agricultural, Food and Environmental Sciences, University of PerugiaPerugia, Italy

**Keywords:** *Arabidopsis thaliana*, flower, sepal, petal, host defense, biotrophic pathogen, salicylic acid

## Abstract

We investigated whether the Arabidopsis flower evolved protective measures to increase reproductive success. Firstly, analyses of available transcriptome data show that the most highly expressed transcripts in the closed sepal (stage 12) are enriched in genes with roles in responses to chemical stimuli and cellular metabolic processes. At stage 15, there is enrichment in transcripts with a role in responses to biotic stimuli. Comparative analyses between the sepal and petal in the open flower mark an over-representation of transcripts with a role in responses to stress and catalytic activity. Secondly, the content of the biotic defense-associated phytohormone salicylic acid (SA) in sepals and petals is significantly higher than in leaves. To understand whether the high levels of stress responsive transcripts and the higher SA content affect defense, wild-type plants (Col-0) and transgenic plants defective in SA accumulation (*nahG*) were challenged with the biotrophic fungus *Golovinomyces cichoracearum*, the causal agent of powdery mildew, and the necrotrophic fungus *Botrytis cinerea*. *NahG* leaves were more sensitive than those of Col-0, suggesting that in leaves SA has a role in the defense against biotrophs. In contrast, sepals and petals of both genotypes were resistant to *G. cichoracearum*, indicating that in the flower, resistance to the biotrophic pathogen is not critically dependent on SA, but likely dependent on the up-regulation of stress-responsive genes. Since sepals and petals of both genotypes are equally susceptible to *B. cinerea*, we conclude that neither stress-response genes nor increased SA accumulation offers protection against the necrotrophic pathogen. These results are interpreted in the light of the distinctive role of the flower and we propose that in the early stages, the sepal may act as a chemical defense barrier of the developing reproductive structures against biotrophic pathogens.

## Introduction

Successful plant sexual reproduction relies on many factors including optimal time of flowering. Consequently, mechanisms have evolved that integrate environmental signals such as light and temperature, with endogenous developmental signals such as autonomous and gibberellin-dependent pathways to regulate flowering time (Simpson and Dean, 2002). However, plants exposed to adverse environmental conditions can activate the flowering program prematurely. Stress factors that are able to promote flowering include pathogen attacks, high levels of ultraviolet light, poor nutrition and drought stresses (Martinez et al., 2004; Kolar and Senkova, 2008; Riboni et al., 2013). It has also been reported that salicylic acid (SA) regulates flowering in Arabidopsis, likely acting as a link between stress responses and the regulation of reproductive development (Martinez et al., 2004). SA-mediated floral promotion appears to be regulated through sumoylation and involves chromatin modifications (Jin et al., 2008). Indeed, SA-dependent regulation of chromatin modification through histone replacement mechanisms may be responsible for maintaining a concomitant repressive state of both systemic acquired resistance to pathogens and transition to flowering in Arabidopsis (March-Diaz et al., 2008). However, the molecular mechanisms of SA-dependent flowering induction remain elusive and little attention has been given to the accumulation of SA in floral organs nor indeed to the function of elevated SA levels, e.g., in the response to microbial pathogens (e.g., Thomma et al., 1998; Noutoshi et al., 2012).

Given that the flower is the organ of sexual reproduction, it is reasonable to expect that this organ has evolved effective morphological structures and mechanisms to protect itself from pathogens. There is growing interest in morphological structures, compounds and mechanisms of biotic defense of the flower (e.g., Rodrigues Marques et al., 2014). However, despite the fact that work in the Arabidopsis model system does offer the obvious advantages of access to a complete genome and transcriptome data as well as a large mutant collection, defense studies in Arabidopsis flowers have remained scarce, not least because of the comparatively small organ size.

It is somewhat surprising that hardly any attention has been given to the role of the sepal in the defense of plants against pathogens. Exceptions are an early report that states that in tobacco a pathogenesis-related protein (PR-1) accumulates in the sepal (Lotan et al., 1989), and more recently, PR-5 promoter activity was observed in tobacco sepal tips (Kenton et al., 2000). There is no report in the literature that would suggest a role of the sepal in the defense against pathogens and this may well be due to the small size of the floral tissue which in the past has limited molecular and biochemical analyses.

In *Arabidopsis thaliana* the sepals are modified green leaf-like organs that enclose the developing flower. They form the outermost whorl - the calyx - of the flower. Early flower development is divided into 12 stages beginning with the initiation of a floral buttress on the flank of the apical meristem (stage 1) and ending with the rapid extension of the petals to the height of the medial stamen (stage 12) (Smyth et al., 1990). The sepal primordia arise in stage 3 and outgrow the flower primordium (stage 4). Petal and stamen primordia appear at stage 5 and end up completely enclosed by the sepals (Smyth et al., 1990). The Arabidopsis flower organs are arranged in concentric whorls as four sepals, four petals, six stamens and two fused carpels (Bossinger and Smyth, 1996). A distinct feature of the sepals is that they contain cells of vastly different sizes, notably the polyploid giant pavement cells that have arisen through endo-reduplication (Roeder et al., 2010), performing karyokinesis but not cytokinesis. While the function of these pavement cells remains unclear, it has been speculated that they may play a role in the defense against insect predators, prevent water stress, and improve the mechanical properties of the organ (Traas et al., 1998), albeit by unspecified mechanisms.

Here we make use of the Arabidopsis model system to perform a comparative system analysis (Meier and Gehring, 2008; Meier et al., 2010) of the sepal and petal transcriptome with a view to gain insight into aspects of organ specific defense responses against pathogen attack. In addition, we also measure SA in the flower and describe responses of the flower to both biotroph and necrotroph pathogens. Finally, we propose that the sepal with its specific morphological characteristics functions not only as a mechanical but also (bio-)chemical defense shield for the developing reproductive organs.

## Materials and methods

### Plant material

*Arabidopsis thaliana* L. Heynh. wild-type Columbia (Col-0) and transgenic *nahG* plant which is defective in the SA accumulation (Lawton et al., 1995) were used in this study. Seeds were surface-sterilized first in 70% (v/v) ethanol and then in 7% (v/v) sodium hypochlorite with 0.2% (w/v) Triton X-100 for 8 min at room temperature under a sterile laminar flow hood. Seeds were rinsed three times with sterile distilled water and re-suspended in 500 μL sterile distilled water. Plants were grown in soil (Patzer Einheitserde, Manna Italia, Bolzano, Italy) in 10 cm pots in a growth chamber with a 14-h photoperiod, a photosynthetic photon fluence rate of 120 μmol m^−2^ s^−1^, day/night air temperatures of 22°C/20°C, and a relative humidity of 60–75%. The plants were watered by sub-irrigation. All seeds were treated at 4°C for 2 days before moving to the growth environment. For all analyses leaves were sampled from 4 week old plants, whereas sepals and petals were taken from completely open flowers corresponding to development stage 14–15 (Smyth et al., 1990) of 6–7 week old plants.

### Scanning electron microscopy

Plant samples from whole soil grown plants were detached with a dissecting knife and immediately placed on a 6 mm-wide double adhesive and conductive tape (Canemco Inc., Quebec, Canada) that was pre-attached onto the specimen stage. The specimen was examined with a bench-top scanning electronic microscope (NeoScope JCM-5000, Jeol Ltd, Tokyo, Japan) and images were acquired using the software provided by the manufacturer.

### Free and total SA extraction and quantification

For SA quantification, four fully expanded leaves were harvested from 9 individual 4 week old plants of two independent cultivations. Sepal and petal SA quantification was carried out twice, sampling 4 mg for each replicate (approximately 80 sepals or petals). Plant material was quick-frozen with liquid nitrogen and stored at −80°C until processed for SA quantification and fungal DNA quantification. To perform SA extraction and quantification, leaf (500 mg FW) and sepal or petal (4 mg FW) samples were pulverized under liquid N_2_ and homogenized in a mortar with 1.5 mL 90% (v/v) methanol in water. The homogenate was centrifuged at 11,000 g for 5 min and the extraction repeated with 0.5 mL 100% methanol. The recovery was evaluated by adding 2.5 μL of *o*-anisic acid (10 mg mL^−1^) as internal standard in the first extraction mixture. All the data were corrected for SA recovery, which ranged from 85 to 100%. After the two extractions the supernatants were combined and the methanol: water mixtures were evaporated in a speed vacuum concentrator with heat (40°C) (Heto, Heto-Holten, Gydevang, Denmark). To avoid sublimation of SA during solvent evaporation 0.2 M sodium hydroxide was added to combined supernatants before concentration. The residue was resuspended in 1 mL of 5% trichloroacetic acid (TCA), mixed by vortex for 10 min, and divided into two 0.5 mL aliquots. One aliquot was passed through 0.2 μm Millipore filters; then, the sample was partitioned with 1 mL of a 1:1 (vol/vol) mixture of ethyl acetate/cyclopentane containing 1% (vol/vol) isopropanol. The uppermost organic phase containing the free SA was then dried by using speed vacuum concentrator. The dried extract was suspended in 0.2 mL of the HPLC mobile phase [methanol: 2% aqueous acetic acid, 45:55 (v/v)] and free SA content was quantified by HPLC. The amount of total SA was quantified as follows: the TCA re-suspended aliquot after filtration through 0.2 μm Millipore filters was added to 1.25 mL of 8 M HCl and hydrolyzed for 1 h at 90°C to release SA from any acid-labile conjugated forms. The released free SA was then partitioned with 3.25 mL of a 1:1 (vol/vol) mixture of ethyl acetate/cyclopentane containing 1% (vol/vol) isopropanol. The top organic phase was dried by using speed vacuum concentrator, resuspended in 0.2 mL of the HPLC mobile phase and analyzed by HPLC. Analysis of free SA was performed in HPLC (Jasco, Tokyo, Japan) by using a 5 μm C18 column (Luna, 150 mm x 4.6 mm; Phenomenex, Inc., Torrance, CA) with isocratic elution. The SA quantification was obtained with a spectrofluorescence detector (Jasco, Tokyo, Japan) using Ex = 209 nm and Em = 402 nm and SA concentrations were calculated using a linear range of calibration standards from 0 to 100 ng SA.

### *Golovinomyces cichoracearum* inoculation

The inoculum of *Golovinomyces cichoracearum* (D.C.) V.P. Heluta (formerly *Erysiphe cichoracearum* D.C.) (Quaglia et al., 2012) were maintained on tobacco plants cv Havana 425 and refreshed on new tobacco plants 10 days before use for Arabidopsis inoculation. Conidia were harvested from tobacco plants by irrigation with sterile deionized water added with 0.04% (v/v) of the surfactant Tween® 20 [10% (v/v) aqueous solution, Boehringer Mannheim, Germany]. The inoculum concentration was measured by hemocytometer and adjusted to 1 × 10^6^ conidia mL^−1^. For leaf inoculation, 4 week old Arabidopsis plants were sprayed with the conidial suspension using a hand atomizer until run-off. Floral spray inoculation was carried out in the same manner on the whole attached inflorescence from 6 to 7 week old Arabidopsis plants. In addition, we also sprayed detached rosette leaves from 4 week old Arabidopsis plants and sepals and petals taken from flowers at stages 14 and 15 (Smyth et al., 1990). All samples were placed in Petri dishes (9 cm diameter) containing 1.2% sterilized water-agar (WA). Plants and plates were incubated in the growth chamber under the conditions described above. The spray inoculation technique allowed to obtain comparable numbers of conidia per area on the surface of the three organs (0.42 ± 0.06 conidia mm^−2^ on the leaves, 0.40 ± 0.08 conidia mm^−2^ on the sepals, 0.39 ± 0.1 conidia mm^−2^ on the petals). Two independent experiments were performed. Leaves, sepals and petals from both plants and plates were taken after 2 and 4 days post-inoculation (dpi) for evaluation of fungal growth. Moreover, macroscopic disease symptoms were evaluated at 7 dpi.

### *Golovinomyces cichoracearum* growth assessment by light microscopy and genomic DNA quantification

Visualization of *G*. *cichoracearum* structures (mycelia, conidiophores and conidia) in all inoculated samples was carried out under the light microscope after Trypan blue staining (Reuber et al., 1998). Briefly, leaves and sepals were immersed in 96% (v/v) ethanol and placed in a 60°C water bath for 15 min to clear the chlorophyll, then gently rinsed for 5 min with deionized water. Petals, cleared leaves and sepals were stained directly on microscope slides with Trypan blue solution [0.01% Trypan blue (v/v) in lactic acid: phenols: glycerol: water (1:1:1:1 v/v)]. Stained samples were observed using a Carl Zeiss (Jena, Germany) microscope at 5 X magnification. On sepals and petals, fungal colonies were analyzed on the entire organ surface while the central portion of the outer leaf surface (three randomly selected areas of 5.3 mm^2^ each) was chosen for analysis. The total number of colonies, and conidiophores per colony and conidia per colony were counted. Data were subject to one-way (genotype) analysis of variance (ANOVA). The means were compared using Duncan's multiple range test at the 1% significance level.

Genomic DNA from Arabidopsis leaves and sepals was isolated from Col-0 and *nahG* plants spray-inoculated with *G. cichoracearum* at 2 and 4 dpi using NucleoSpin® Plant II kits (Macherey-Nagel, Düren, Germany) according to the manufacturer instructions. Plant and fungal biomass content in the DNA extracts was determined by independent semi-quantitative PCR analysis where the reaction mixtures contained the primer pair designed for the Arabidopsis gene AT5G19510 (elongation factor; forward 5′-TGATGTCAAGGTTTACGCTG-3′ and reverse 5′-ACTCTCTTTAGGCTTCTTGG-3′), or primer sequences specific for *G*. *cichoracearum* were derived from the ribosomal ITS region of the fungus (forward 5′-GGTTGTGTCCGCCAGAGACC-3 and reverse 5′-TGATGTCAAGGTTTACGCTG-3) as reported elsewhere (Chen et al., 2008). Cycling parameters were as follows: initial denaturation at 94°C for 2 min, followed by 28 cycles of 1 min at 94°C, 1 min at 58°C, 1.5 min at 72°C, and final extension at 72°C for 5 min. Serial dilutions of pure genomic DNA from *G. cichoracearum* and Arabidopsis were used to trace a calibration curve that was used to quantify plant and fungal DNA in each sample. Results are presented as a ratio between fungal and plant DNA in the leaf and sepal. Each data point represents the mean of two independent biological samples.

### *Botrytis cinerea* inoculation and lesion evaluation

An isolate of *Botrytis cinerea* Pers. ex Fr. (Quaglia et al., 2011) was used essentially as described elsewhere (Muckenschnabel et al., 2002), conidia were harvested by irrigation with a sterile aqueous solution of 10 mM sucrose and 10 mM KH_2_PO_4_ added with 0.04% (v/v) of the surfactant Tween®20 from 10 days old colonies grown on Potato Dextrose Agar, at 21 ± 2°C, in the dark. The spore suspension was passed through two layers of cheese cloth and, after counting the numbers of spores with a hemocytometer, adjusted to the final concentration of 1 × 10^5^ conidia mL^−1^. Inoculation with 5 mL of conidia in suspension was performed by spraying the conidia until run-off on 4 week old leaves or inflorescences of 6–7 week old Arabidopsis plants. This was performed on detached plant organs placed in Petri dishes (9 cm diameter) containing 1.2% WA. The plates were incubated in the growth chamber at the conditions described above and the high humidity was maintained by covering the plastic lid. Spray-inoculated plants were observed at 2 and 7 dpi for qualitative evaluation of the infection. On drop-inoculated detached leaves necrotic lesions area was determined at 3 dpi (Ferrari et al., 2007) and data were subject to one-way (genotype) analysis of variance (ANOVA). The means were compared using Duncan's multiple range test, at the 1% significance level.

### Transcriptomics analyses

Analyses were performed on sepal data (Voelckel et al., 2010) and publicly available microarray data from the AtGenExpress developmental series (Schmid et al., 2005) for flowers and pollen (GSE5632) and leaves (GSE5630) for sepal (stage 15, GSM131603-GSM131605), petal (stage 15, GSM131606-GSM131608), rosette (GSM131510-GSM131512), leaf 1+2 GSM131498-GSM131500) and senescent leaf (GSM131537-GSM131539) samples were downloaded from Gene Expression Omnibus (GEO, http://www.ncbi.nlm.nih.gov/geo/, Edgar et al., 2002; Barrett et al., 2011). Arrays were normalized using Robust Multi-Array Averaging (RMA) in the Bioconductor affylmGUI package (Smyth, 2004; Smyth et al., 2005; Wettenhall et al., 2006) before a linear fit model was applied and the following contrasts (differentials) calculated: sepal vs. petal, sepal vs. rosette, sepal vs. leaf (not shown), sepal vs. senescent leaf, petal vs. senescent leaf. The top 25 most up-regulated genes in each contrast were then selected according to descending B-statistics. GO analyses were performed on each up-regulated gene list using AgriGO (http://bioinfo.cau.edu.cn/agriGO/, Du et al., 2010). The data from the transcriptional analyses are in Table S1.

## Results

### Morphological characteristics of the *Arabidopsis thaliana* sepal

**Figure 1:**
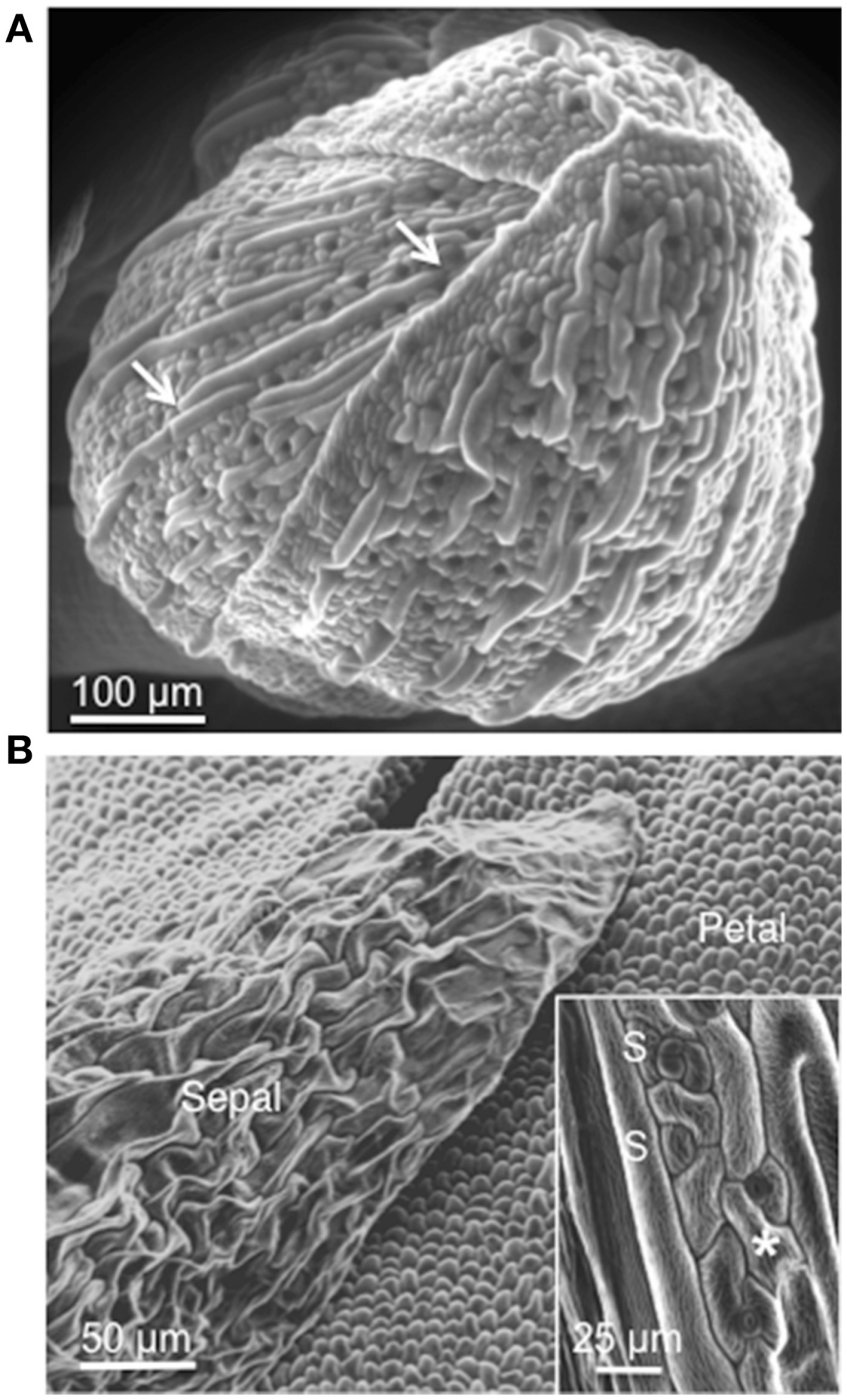
**Scanning electron micrograph (SEM) of an Arabidopsis flower. (A)** Young developing bud (stage 9–12) showing abaxial (outer) side of sepals. Sepals are bent and the upper edges are overlapping and appear tightly sealed. White arrows indicate giant cells that are interspersed between smaller cells. **(B)** Open bud (stage 14–15). Abaxial side of sepals and petals are visible. The inset in **(B)** shows epidermal cells of sepal characterized by some interdigitation (asterisk) and stomata (S).

In Arabidopsis the sepals are green leaves, not dissimilar to upper shoot vegetative leaves, that enclose the developing reproductive organs. Scanning electron microscopy reveals that in the young developing buds, prior to the emergence of the petals, the leaves are bent and the upper edges are overlapping and appear tightly sealed (Figure 1A) effectively completely enclosing the reproductive organs. The pavement cells of the sepal show a degree of interdigitation (Figure 1B, inset), albeit less pronounced than in mature vegetative leaf cells. Separated by rows of ≥2 pavement cells of “normal” size, we find highly elongated giant cells with hardly any interdigitation typical for leaf epidermal cells (Staff et al., 2012). The sepal is also characterized by a high number of stomata (≥3/100 μm^2^) on the upper surface, which is directly exposed to the atmosphere, indicating high metabolic activity but also vulnerability to pathogens that can enter through the stomata (Melotto et al., 2008; Zhang et al., 2008) and may do so by forcing stomata open (Gottig et al., 2008). Bud burst is characterized by the breaking of the sepal seal and emergence of the petals (Figure 1B). The latter show small cells that are more elongated at the base and more globular at the tip of the growth axis and do not contain stomata. By this stage, the sepal seems to have largely lost the mechanical protective function and we note a loss of turgor and a decrease in cellular organization indicative for the onset of senescence (Warner et al., 2000).

### Inferences from the sepal transcriptome

In order to gain insight into the functions of the sepal, a systems level analysis of transcriptional data from different stages was undertaken (Meier et al., 2008, 2010; Meier and Gehring, 2008). Of the 25 most abundant transcripts at stage 12 (Table 1), a significant enrichment of genes in the gene ontology (GO) categories “Response to chemical stimulus” (RCS) and “Cellular metabolic process” (CMP) was noted. Furthermore, the *SA UDP-glucosyltransferase* (AT2G43820) involved in the formation of SA 2-O-β-glucoside (SAG) and its glucose ester (SGE) is one of the 10 most expressed genes at this stage. Dramatic increases in both these conjugates are considered a hallmark of the biotic defense program.

**Table 1:** **Most expressed sepal genes at stage 12**.

**Gene ID**	**GO cat**.	**Annotation**
AT4G25100	RCS CMP	Superoxide dismutase
AT3G62380		Hypothetical protein
AT1G05680		UDP-glucosyltransferase, acts on IBA
AT2G25510		Expressed protein
AT2G21220	RCS	Auxin-responsive protein
AT5G43450		2-Oxoglutarate-dependent dioxygenase
AT5G14740		Carbonic anhydrase 2
AT5G24150		Squalene monooxygenase 11
AT2G43820	^*^	SA UDPglucosyltransferase—SAGT (SA ↓)
AT1G05560	RCS CMP	UDP-gluc. transfer. 1 (ABA)
AT5G22300	RCS CMP	Nitrilase 4 (NIT4)
AT4G26530	CMP	Fructose-bisphosphate aldolase
AT1G14150	CMP	O_2_ evolving enhancer 3
AT3G28220		Meprin and TRAF homol. domain protein
AT2G41090		CAM-like binding protein
AT4G17340		MIP family protein, TIP2;2
AT5G58770	CMP	Dehydrodolichyl diphosphatase synthase
AT3G14630		Cytochrome P450 putative
AT1G23130		Bet v I allergen
AT3G22340		Copia-like retro-transposon
AT1G59870	RCS CMP	ABC transporter protein, ABCG36
AT3G03480	CMP	Acetyl CoA:(Z)-3-hexen-1-ol acetyl transf.
AT3G57230	CMP	MADS-box protein
AT3G01290		Band 7 family protein
AT4G23600	RCS CMP	Coronatine-ind., JA and ABA resp. Cys lyase

RCS, Response to chemical stimulus (GO: 0051707, FDR: 0.04); CMP, Cellular metabolic process (GO: 0044237, FDR: 0.02);

* *Salicylic acid response*.

At stage 15 when the stigma extends above the anthers, we find that the 25 most highly expressed genes (Table 2) are enriched in the categories “Response to biotic stimulus” (RBS), “Response to chemical stimulus” (RCS), and “Response to other organisms” (ROO) and nine of the most highly expressed genes encode proteins that occur in the extracellular space. The functional enrichment is even more pronounced in the 10 most expressed genes (Table 2), with half of the encoded proteins being targeted to the extracellular space and three directly involved in biotic stress responses (GO:0009607). One of the genes not directly involved in stress responses is the Agamous-like 16 MADS-box encoding gene that is expressed in leaf, root and stem, with higher RNA accumulation in guard cells and trichomes. This protein is believed to have a role in stomatal lineage progression and stomatal development (Kutter et al., 2007) and is induced in response to ABA and by pathogens.

**Table 2:** **Most expressed sepal genes at stage 15**.

**Gene ID**	**GO cat**.	**Annotation**
AT2G43570	#	Chitinase
AT2G14610	# RBS RCS ROO	Pathogenesis-related protein 1 (PR-1)
AT3G57230		MADS-box protein (AGL16)
AT4G25100	RCS	Superoxide dismutase [Fe]chloroplast
AT1G75040	# RBS ROO	Pathogenesis-related protein 5 (PR-5)
AT2G14560	RBS RCS ROO	Coronatine-induced protein
AT4G23600	# RCS	Coronatine-ind., JA and ABA res. Cys lyase
AT1G35710		Leucine-rich repeat transmembrane kinase (LRRK)
AT2G18660	#	Plant Natriuretic Peptide (AtPNP-A)
AT3G01290		Band 7 family protein, myristoylation
AT4G11650	# RBS ROO	Osmotin-like protein (OSM34), biotic defense
AT5G10760	#	Aspartyl protease family protein
AT2G43820	^*^	SA UDPglucosyltransferase—SAGT (SA ↓)
AT5G22300	RCS	Nitrilase 4 (NIT4)
AT3G60390		Homeobox-leucine zipper protein 3 (HAT3)
AT3G50420		Pentatricopeptide (PPR) repeat protein
AT4G14365		Zinc finger/ankyrin repeat family protein
AT1G61800	RBS RCS ROO	Glucose-6-phosphate/phosphate translocator
AT1G21250	# RCS	Wall-associated kinase 1 (WAK1)
AT3G22550		Senescence-associated protein
AT1G20070		Expressed chloroplast protein
AT5G23010		2-Isopropylmalate synthase 3 (IMS3)
AT2G44240	# RCS	Oxidative stress response protein
AT4G37370		Cytochrome P450 putative
AT3G28500		60S Acidic ribosomal protein P2 (RPP2C)

RBS, Response to biotic stimulus (GO: 0009607, FDR: 0.0009); RCS, Response to chemical stimulus (GO: 0042221, FDR: 0.0009); ROO, Response to other organisms (GO: 0051707, FDR: 0.0009);

* Salicylic acid response;

# *Proteins directed to the extracellular region, apoplast*.

Another of the highly expressed genes encodes a leucine-rich receptor kinase (LRRK; AT1G35710). If we do a GO analysis on the top 25 expression correlated genes (*r* > 0.85) with AT1G35710, we observe that they are highly significantly enriched in the categories “Defense response” (GO:0006952; FDR = 9.8e^−08^) and “Response to salicylic acid stimulus” (GO: 0009751; FDR = 7.6e^−06^). From this we inferred that the protein is involved in responses against pathogen defense and this is again entirely consistent with its stimulus specific expression profile (Zimmermann et al., 2004).

Furthermore, a gene encoding a Band 7 family protein that is involved in N-terminal myristoylation, the modification of a protein with a hydrophobic 14-carbon fatty acid myristate that enables membrane attachment of soluble proteins, protein targeting and interactions and partitioning into specific membrane domains (Sorek et al., 2009) is also highly expressed. This is relevant since post-translational protein modifications including myristoylation have a role in pathogen-induced defense signaling (Stulemeijer and Joosten, 2008) and can both specifically activate (Nimchuk et al., 2000; Shan et al., 2000) or repress (Andriotis and Rathjen, 2006) defense signaling components.

Also in the group of the 10 most abundant sepal transcripts is *AtPNP-A* (AT2G18660) that encodes a Plant Natriuretic Peptide with a role in both responses to pathogens (Gottig et al., 2008; Meier et al., 2008) as well as photosynthesis and the regulation of cellular homeostasis (Garavaglia et al., 2010; de Jonge et al., 2012). In addition, both a chitinase (AT2G43570) and an aspartyl protease family protein (AT5G10760) are annotated as targeted to the extracellular space where they are likely to act in the chemical defense against pathogens.

Another abundant transcript encodes a nitrilase (NIT4; AT5G22300) that acts on C-N bonds (but not peptide bonds). The *NIT4* expression profile appears to be highly development- and organ-specific, suggesting a critical role in the sepal. Expression is strongly down-regulated in response to methyl-jasmonate and strongly induced by *Pseudomonas parasitica* and *Pseudomonas syringae*, but not in *penta* mutants (loss-of-function in the gibberellic acid (GA) mutants GAI, RGA, RGL1, and RGL2, four DELLA genes) where expression is suppressed. The expression data would therefore support the idea that GA can signal through jasmonates in flower development (Cheng et al., 2009).

In a next step we analyzed the promoters of the highly expressed genes in the sepal. In the top 10 (stage 15) we find a significant enrichment (*P* < 10^−4^) of the LS7 element (ACGTCATAGA). The *cis*-element is found in the promoter of the *PR-1* gene and in the coronatine-induced gene (Table 2). Previously the promoter of *PR-1* has been reported to contain two putative TGA transcription factor-binding targets termed *linker scan7* (*LS7*) and *LS5* (Lebel et al., 1998), the former acting as positive regulator of *PR-1* expression in response to 2,6-dichloroisonicotinic acid and SA. The TGA transcription factors regulate expression of *PR* genes through their interaction with the positive regulator NPR1 (non-expresser of *PR*-*1*). In particular, *PR-1* expression was reported to be dependent on TGA factor recruitment to the LS7-containing *PR-1* promoter in an SA- and NPR1-dependent manner (Johnson et al., 2003).

If we expand the promoter content analyses to the 100 most highly expressed sepal genes, we find significant enrichments for the I-box motif (*P* < 10^−4^) and the W-box motif (TTGACC or TTGACT; *P* < 10^−4^) that is the target site for WRKY transcription factors which in turn have been implicated in the regulation of transcriptional re-programming associated with plant immune responses (Eulgem and Somssich, 2007). *NPR1* expression is important for the activation of plant defense responses and WRKY encoding genes act upstream of *NPR1* and promote its expression during the activation of plant defense responses, a mechanism entirely consistent with SA-induced expression of *WRKY* (Yu et al., 2001). In addition, it was noted that five *WRKYs* (AT2G30250, AT4G01720, AT2G23320, AT5G07100, and AT4G31550) are positively expression correlated (*r* > 0.65) with the *Band 7 family protein* that has a role in myristoylation which in turn is implicated in pathogen-induced defense signaling (Stulemeijer and Joosten, 2008).

Since pathogen defense is likely to be an essential function of the sepals, we were interested to see the induction profile of the isochorismate synthase (*ICS*) gene (AT1G74710) that is a key gene for SA biosynthesis in Arabidopsis. Between stages 12 and 15 it increased >1.5-fold. Remarkably, one of the highly expressed genes at the stages 12 and 15 encodes a SA UDP-glucosyltransferase (AT2G43820) that inhibits the accumulation of SA (Tables 1, 2). Recently, it has been demonstrated that activators of defense that inhibit SA glucosyltransferases (SAGTs) can indeed augment the pool of free SA and thus enhance plant resistance to pathogens (Noutoshi et al., 2012). It would therefore appear that the synthesis of SA is not only enhanced in the sepal but also very tightly controlled.

### The sepal, petal, and rosette leaf have specific transcriptomic signatures

In order to gain further insight into unique sepal functions, we have undertaken to identify transcripts that are differentially expressed in the sepal as compared to the petal and/or rosette leaves. The comparison between the highly up-regulated genes in the sepal as compared to the petal revealed that in this group the top 25 differentially expressed genes are enriched in the categories “Response to stress” and “Catalytic activity” (Table 3 and Table S1). This is consistent with a specialized role in defense and in chemical defense in particular given that the latter depends heavily on catalytic activity essential for the production of flavonoids, phenolics, glucosinolates, terpenoids, and alkaloids (Kliebenstein, 2012). When the highly expressed sepal genes were compared to the rosette leaf transcriptome, a significant enrichment for proteins with a role in catalysis was observed (Table 4 and Table S1). Again, enhanced catalytic activity is an indication of chemical defense. Since the senescence response shares some similarity with the defense response, we also looked at highly up-regulated genes in the sepal rather than in the senescent leaf and found significant enrichments in the categories “Response to external stimulus” and “Response to endogenous stimulus” (Table 5 and Table S1). These responses are defined as any process that results in a change in the state or activity of a cell or organism, e.g., in terms of secretion or enzyme production as a result of an external stimulus. The result therefore is further support for a sepal specific metabolic response different to the one observed in leaf senescence. Finally, a similar comparison between the petal and the senescent leaf transcriptomes sees the categories “Cell wall” and “External encapsulating structures” enriched in the petal (Table 6 and Table S1).

**Table 3:** **Genes highly up-regulated at stage 15 sepals vs. petals**.

**Gene ID**	**GO cat**.	**Annotation**
AT4G17030		EG45-like domain containing protein 2
AT4G25100	RS CA	Superoxide dismutase [Fe]
AT1G02920	CA	Glutathione S-transferase 11
AT4G23600	RS CA	Coronatine-induced, JA and ABA responsive
AT1G19580	CA	Carbonic anhydrase, chloroplastic
AT2G43570	CA	Chitin-binding, chitinase activity
AT3G13790	RS CA	β-Fructofuranosidase, insoluble isoenz.
AT4G23150	CA	Cysteine-rich receptor-like protein kinase 7
AT2G02930	CA	Glutathione S-transferase 16
AT2G37770	CA	NADPH-dependent aldo-keto reductase
AT1G75040	RS	Pathogenesis-related protein 5 (PR-5)
AT3G23110	RS CA	Receptor-like protein 37, defense response
AT5G19440		Alcohol dehydrogenase, NAD activity
AT1G52200	RS	Divalent metal ion transport
AT3G51600		Lipid transfer protein (PR-14) family
AT3G23570	CA	α/β-Hydrolases superfamily protein, salt resp.
AT3G01290		Defense response to fungus
AT2G05380		Glycine-rich protein 3
AT4G14365		XB3 ortholog 4, defense (zinc-finger protein)
AT5G23010	CA	2-Isopropylmalate synthase 3 (MAM1)
AT1G13080	RS	Cytochrome P450 71B15
AT3G57260	RS CA	β1,3-Glucanase
AT3G22600		Lipid-transfer protein
AT5G44580		Regulator of defense response (SAR)
AT2G26440	CA	Pectinesterase/pectinesterase inhibitor 12

*RS, Response to stress (GO: 0006950, FDR: 0.001); CA, Catalytic activity (GO: GO:0003824, FDR: 0.0023)*.

**Table 4:** **Genes highly up-regulated at stage 15 sepal vs. rosette leaves**.

**Gene ID**	**GO cat**.	**Annotation**
AT2G38540		Non-spec. lipid transfer prot., binds CAM
AT1G35310		MLP-like protein, defense response
AT5G45890	CA	Senescence-assoc. gene 12 (Cys-type pep.)
AT2G37770	CA	NADPH-dependent aldo-keto reductase
AT3G13400		Multicopper oxidase
AT1G68620	CA	Hydrolase superfamily protein
AT2G02990	CA	Ribonuclease 1
AT4G24000	CA	Cellulose synthase G2
AT4G23680		Polyketide cyclase, lipid transport
AT1G02790	CA	Exopolygalacturonase
AT4G15620		UPF 497 membrane protein
AT3G27810		MYB21, R2R3-MYB family
AT1G80160	CA	Lactoylglutathione lyase
AT5G15800		Developmental protein SEPALLATA 1
AT1G54570	CA	Acyltransferase-like protein, chloroplast
AT2G47030	CA	Pectinesterase 4
AT4G33040	CA	Glutaredoxin-C6
AT5G02580		Unknown protein
AT1G61563		Rapid Alkalinisation Factor 8
AT2G41380	CA	S-adenosyl-L-met.-dep. methyl transferase
AT1G09500	CA	Alcohol dehydrogenase
AT1G65480		Flowering locus T, promotes flowering
AT1G61680	CA	Linalool synthase, chloroplastic
AT4G39480		Cytochrome p450, family 96 protein
AT5G07430	CA	Pectin lyase-like superfamily protein

*CA, Catalytic activity (GO: 0003824, FDR: 0.004)*.

**Table 5:** **Genes highly up-regulated at stage 15 sepals vs. senescent leaves**.

**Gene ID**	**GO cat**.	**Annotation**
AT1G35310		MLP-like protein, defense response
AT2G38540		Non-spec. lipid transfer prot., binds CAM
AT1G19580		Carbonic anhydrase, chloroplastic
AT5G59310	REN	Lipid-transfer prot. 4, abiotic stress
AT1G55260		Lipid-transfer protein
AT5G24150		Squalene monooxygenase 1,1
AT5G15800		Developmental protein SEPALLATA 1
AT3G27810	REN	MYB21, R2R3-MYB family
AT1G65480		Flowering locus T, promotes flowering
AT4G15210		Cytosolic β-amylase
AT1G02205		Production of stem epicuticular wax
AT1G61680		Linalool synthase, chloroplastic
AT4G14690		Early light-induced protein. ELIP
AT1G69120		Apetala 1
AT1G29670		GDSL-like lipase
AT1G24260		MADs box transcription factor
AT4G39480		Cytochrome p450, family 96 protein
AT2G02990	REX REN	Ribonuclease 1
AT1G66120		Butyrate metabolic process
AT4G23600	REX REN	Coronatine-ind., JA and ABA resp.
AT2G37770		NADPH-dependent aldo-keto reductase
AT3G11480	REX	Methyltransferase for SA and benzoic acid
AT2G06850	REX REN	Xylogluc. endotransglucosylase/hydrol.
AT5G57560	REX REN	Xylogluc. endotransglucosylase/hydrol.
AT1G35140		EXL1 is involved in the C-starvation

*REX, Response to external stimulus (GO: 0009605, FDR: 0.00045); REN, Response to endogenous stimulus (GO: 0009719, FDR: 0.0017)*.

**Table 6:** **Genes highly up-regulated at stage 15 petals vs. senescent leaves**.

**Gene ID**	**GO cat**.	**Annotation**
AT5G25460	CW EXE	DUF 642, plant-type cell wall function
AT1G61680		Linalool synthase, chloroplastic
AT3G27810		MYB 21, R2R3-MYB family
AT1G55260		Lipid-transfer protein
AT2G06850	CW EXE	Xylogluc. endotransglucosylase/hydrolase
AT1G29670	CW EXE	GDSL-like lipase
AT2G10940		Lipid-transfer protein
AT2G38540	CW EXE	Non-spec. lipid transfer prot., binds CAM
AT1G35310		MLP-like protein, defense response
AT4G39480		Cytochrome p 450, family 96 protein
AT3G53300		Putative cytochrome p 450
AT1G02205		Production of stem epicuticular wax
AT1G66120		Butyrate metabolic process
AT3G54340		Apetala3
AT3G01980		NAD(P)-bind. Rossmann-fold protein
AT1G55330		Arabinogalactan peptide 21
AT2G17880		Chaperone DnaJ-domain superfam. prot.
AT5G45950		GDSL-like Lipase
AT4G32460	CW EXE	Unknown protein in the cell wall
AT5G62360	CW^*^	Unknown protein in the cell wall
AT4G25830		UPF0497 membrane protein
AT5G47550	CW EXE	Cysteine proteinase inhibitor 5
AT1G12090		Extensin-like protein (ELP)
AT1G24260		Sepallata 3
AT1G11850		Unknown protein

CW, Cell wall (GO: 000 5618, FDR: 4.5e^−06^); EXE, External encapsulating structure (GO: 0030312, FDR: 4.5e^−06^);

* *Not included in the AgriGO analysis*.

### The flower shows elevated levels of salicylic acid

**Figure 2:**
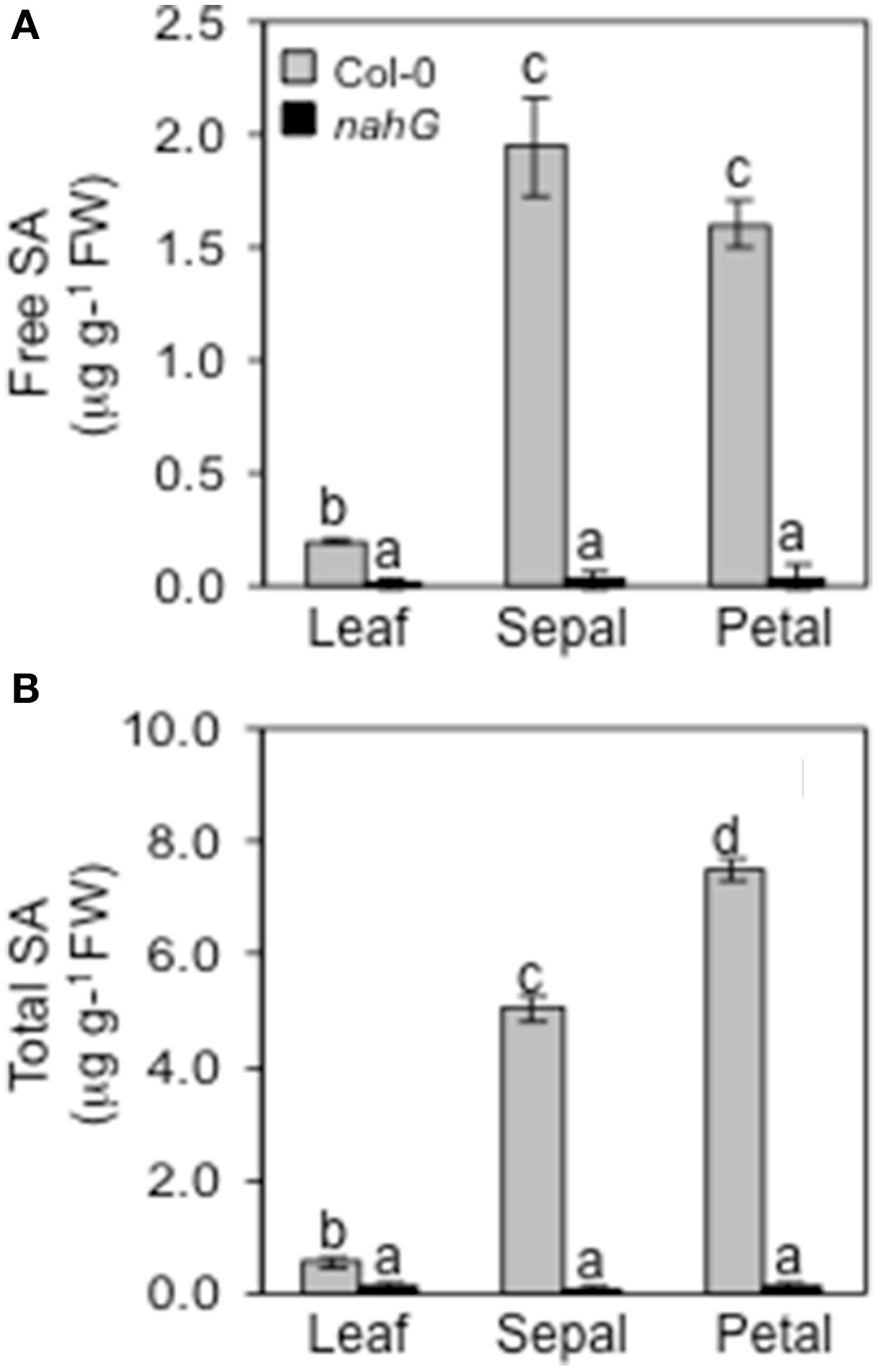
**Endogenous levels of free and total SA**. Free **(A)** and total **(B)** endogenous SA content in leaves, sepals and petals of Col-0 and *nahG* plants were determined. Leaves were sampled from 4 week old plants, whereas sepals and petals were taken from 6 to 7 week old plants at stage 14–15 of flower development. Bars represent the mean ± SE and different letters indicate statistically significant differences using Duncan's multiple range test (*P* ≤ 0.01).

Given that the transcriptome analysis showed that the SA-induced *PR-1* was over expressed in sepals in stage 15 (Table 2), we were interested to discover if SA accumulates in sepals and petals. We assessed the total (free and sugar-conjugated) and free SA levels in leaves, sepals and petals of Arabidopsis Col-0. The glucosylated form makes the largest part of the total SA content. In the leaves it is 64%, in the sepal 62 and 79% in the petal (Figure 2). It is noteworthy that free and total SA content were significantly higher in sepals as compared to leaves (≈10-fold). In petals the free SA levels were similar to those in the sepals while the total SA was considerably higher (Figure 2A). We also performed experiments to detect the levels of total and free SA in leaves and in floral organs of plants carrying the *nahG* transgene salicylate hydroxylase that converts SA to catechol. As expected, *nahG* plants accumulated just detectable quantities of total and free SA without any significant differences among the three organs examined (Figure 2).

### Plant responses to infection with *Golovinomyces cichoracearum*

**Figure 3:**
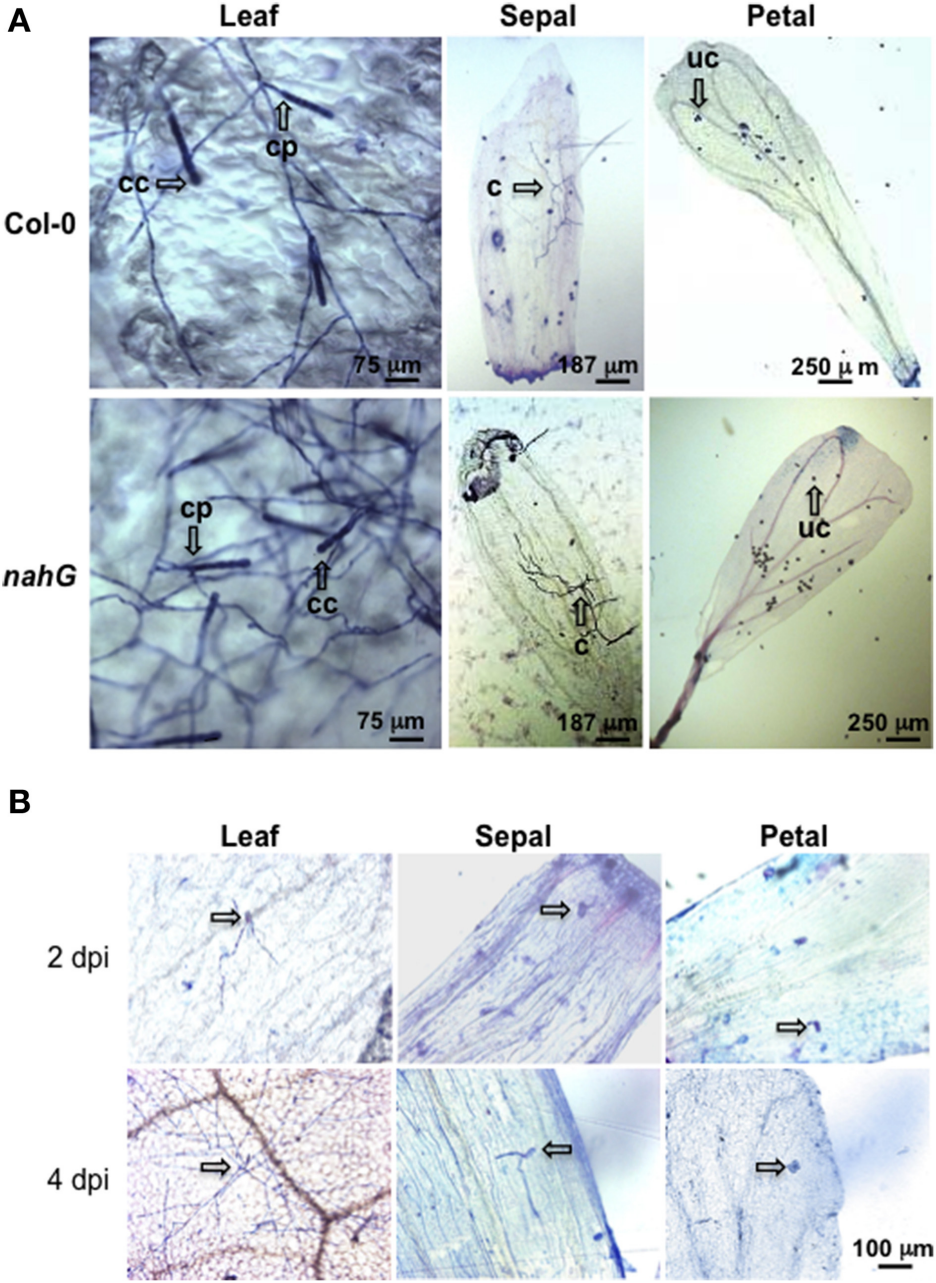
**Trypan blue staining for the detection of *Golovinomyces cichoracearum*. (A)** Representative microscopic images of leaves, sepals and petals from Col-0 and *nahG* plants stained with Trypan blue at 4 days post-spray inoculation with a conidial suspension of *G. cichoracearum*. Leaves were sampled from 4 week old plants, whereas sepals and petals were taken from 6 to 7 week old plants at stages 14–15. Observations were carried out on a minimum of 50 samples. Arrows indicate a conidiophore (cp) and chain of conidia (cc) in leaves, colony without conidiphore (c) in sepals and ungerminated conidia (uc) in petals. **(B)** Representative microscopic images of detached leaves, sepals and petals from Col-0 stained with Trypan blue at 2 and 4 days post-spray inoculation with a conidial suspension of *G. cichoracearum*. Arrows indicates developed colonies in leaves and ungerminated or just germinated conidia in sepals and petals.

**Figure 4:**
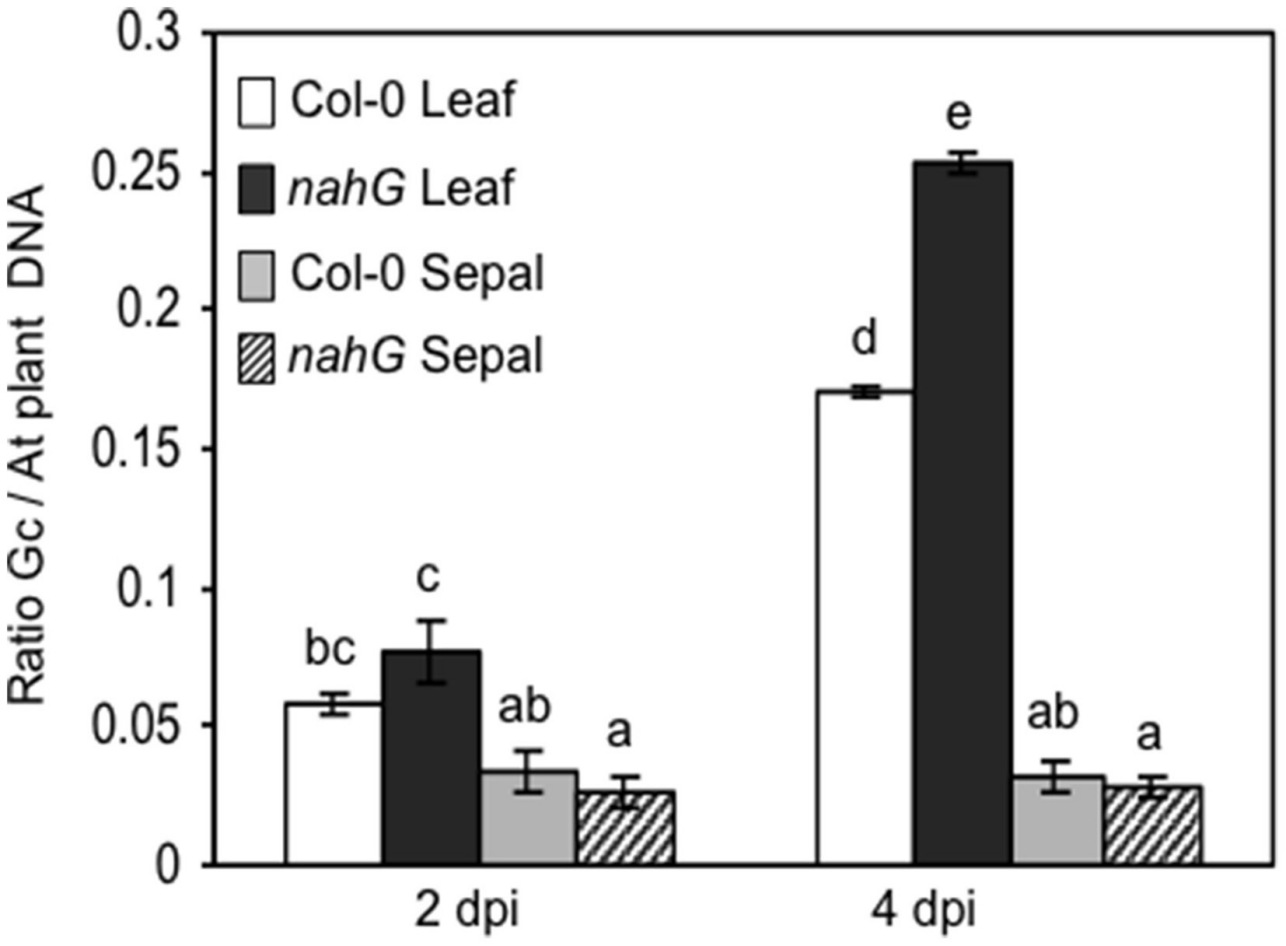
**Genomic DNA quantification of *Golovinomyces cichoracearum* biomass**. *G. cichoracearum* biomass was quantified by semi-quantitative PCR in leaves and sepals of Col-0 and *nahG* plants at 2 and 4 dpi and expressed as the ratio between fungal and plant DNA. Leaves were sampled from 4 week plants, whereas sepals and petals were taken from 6 to 7 week old plants, at stage 14–15. Amplification was performed using specific primers derived from the ribosomal ITS region of the fungus and primers designed for the Arabidopsis elongation factor (AT5G19510). Bars represent the mean ± SE of two biological replicates (a pool of leaves or sepals from 10 plants per replicate) analyzed in triplicate by PCR assay. Two-way (genotype and time) analysis of variance was performed. Different letters indicate statistically significant differences using Duncan's multiple range test (*P* ≤ 0.01).

The markedly higher SA content in Col-0 sepals and petals compared to leaves might suggest a different defense reaction of these organs against biotrophic pathogens. To test this, Col-0 and SA-deficient *nahG* plants were inoculated with *G. cichoracearum*. At 4 days post-inoculation (dpi) all leaves of both genotypes were infected, as seen from the count of the Trypan blue stained colonies (Figure 3A and Table 7). However, on SA-deficient *nahG* leaves, the pathogen produced significantly more conidiophores and conidia per colony than on the Col-0 leaves (Table 7). In contrast, at the same time (4 dpi) only on a very few sepals did *G. cichoracearum* form colonies and they did not develop conidiophores and conidia. In general, the colony growth on sepals appeared to be impaired in comparison to the leaf (Figure 3A). At 4 dpi, no colonies were detected on inoculated petals in both Col-0 and *nahG* genotypes (Figure 3). We also carried out the *G. cichoracearum* inoculation on detached Col-0 rosette leaves, sepals and petals incubated in Petri dishes on water-agar medium. At 2 dpi, only a small number of colonies were found on leaves, while the number of colonies was significantly higher at 4 dpi. At 4 dpi, only a single colony was detected on all sepals examined (Figure 3B) and no germinated conidia were found on petal surfaces (Figure 3B). Similar results were obtained in the floral organs detached from *nahG* plants. Moreover, at 4 dpi in the leaves of the mutant fungal colonies were more developed with respect to those detected on Col-0 (Figure S1). The results of *G. cichoracearum* infection quantification are reported in Table 7 and Figure 4. To further assess the level of *G. cichoracearum* infection on Col-0 and *nahG* leaves and sepals, the fungal biomass was quantified by PCR analysis at 2 and 4 dpi. At 2 dpi fungal biomass was similar in the leaves of both genotypes whereas at 4 dpi, *G. cichoracearum* biomass was significantly higher in *nahG* leaves (Figure 4). On Col-0 and *nahG* sepals, the fungal biomass was similar and significantly lower than that of the leaves; moreover, it did not increase at 4 dpi (Figure 4). The genomic DNA quantification of *G. cichoracearum* was restricted to the leaves and sepals since the petals were never infected by *G. cichoracearum*.

**Table 7:** **Quantitation of *Golovinomyces cichoracearum* growth on Col-0 and *nahG* leaves and sepals**.

**Genotype**	**Examined leaves**	**Leaves with colonies (%)**	**Total colonies**	**Conidiophores/Colony**	**Conidia/Colony**
Col-0	24	100	98	0.64 ± 0.13^a^	2.13 ± 0.61^a^
*nahG*	24	100	284	1.94 ± 0.22^b^	5.40 ± 0.71^b^
**Genotype**	**Examined sepals**	**Sepals with colonies (%)**	**Total colonies**	**Conidiophores/Colony**	**Conidia/Colony**
Col-0	60	6	5	0	0
*nahG*	60	5	6	0	0

*Leaves and sepals of Col-0 and nahG plants were sprayed with a conidial suspension of G. cichoracearum. Leaves were sampled from 4 week old plants, whereas sepals and petals were taken from 6 to 7 week old plants at flower stages 14–15. Samples were stained with Trypan blue at 4 dpi. Experiments were repeated 3 times with similar results. Conidiophores and conidia were counted on randomly selected single fungal colonies on a total of 24 leaves and 60 sepals per genotype. Data represent the mean ± SE. Different letters indicate statistically significant differences using Duncan's multiple range test (P ≤ 0.01)*.

**Figure 5:**
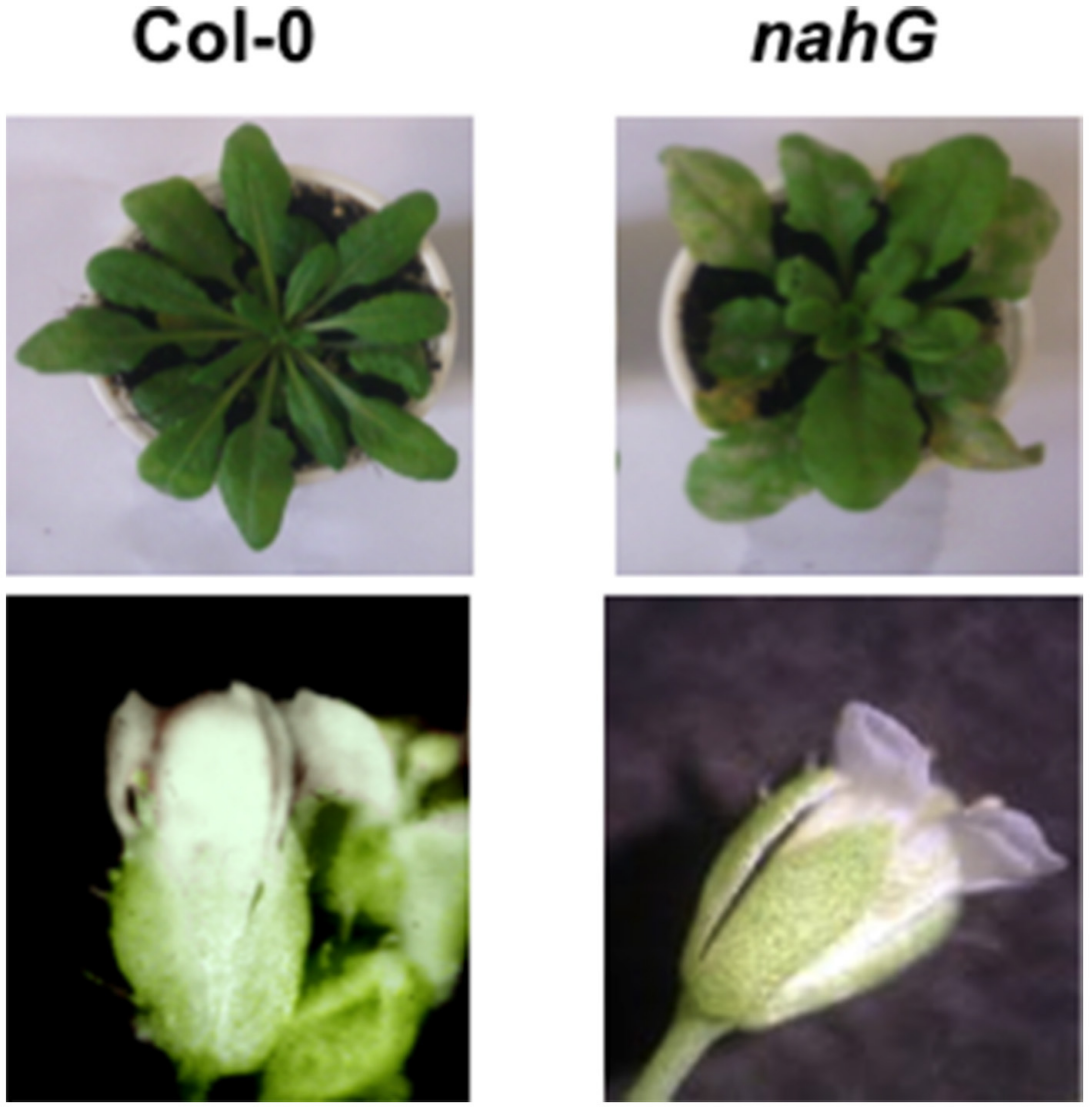
**Symptoms caused by the biotrophic pathogen *Golovinomyces cichoracearum***. Chlorosis and chlorosis plus necrosis were visualized 7 dpi on Arabidopsis Col-0 and *nahG* leaves sprayed with a conidial suspension of *G. cichoracearum*. On *nahG* leaves a typical white powdery was observable. At the same time, symptoms of infection were not detected on Arabidopsis Col-0 and *nahG* inoculated flowers. The experiment was repeated 3 times with similar results. Ten replicates (leaf rosette or inflorescence) per experiment were used.

Usually, on infected host surfaces, *G. cichoracearum* shows abundant conidiation that is visible to the naked eye, starting 7 dpi, even though there is wide variability in infection phenotypes in terms of isolate virulence, environmental conditions (such as temperature, humidity and light intensity) and host susceptibility. Here, visual assessment of inoculated plants at 7 dpi revealed chlorotic and necrotic lesions on *nahG* leaves and the leaf surfaces had the white powdery appearance caused by mycelia, conidiphores and conidia. At the same time, only small chlorotic lesions and limited conidiation were detectable on Col-0 leaves. In contrast, no disease symptoms or pathogen structures were detectable on flowers of both of these genotypes (Figure 5). On the other hand, our microscopic investigation (Figure 3) clearly showed that at 4 dpi the spray-inoculated conidia had not germinated or only just germinated on the surface of the floral organs, without development of fungal hyphae. The fungal DNA quantification confirmed an absence of growth of the fungus on Col-0 and *nahG* sepals (Figure 4), which indicated that these reproductive structures are resistant to *G. cichoracearum* infection.

### Plant responses to infection with *Botrytis cinerea*

**Figure 6:**
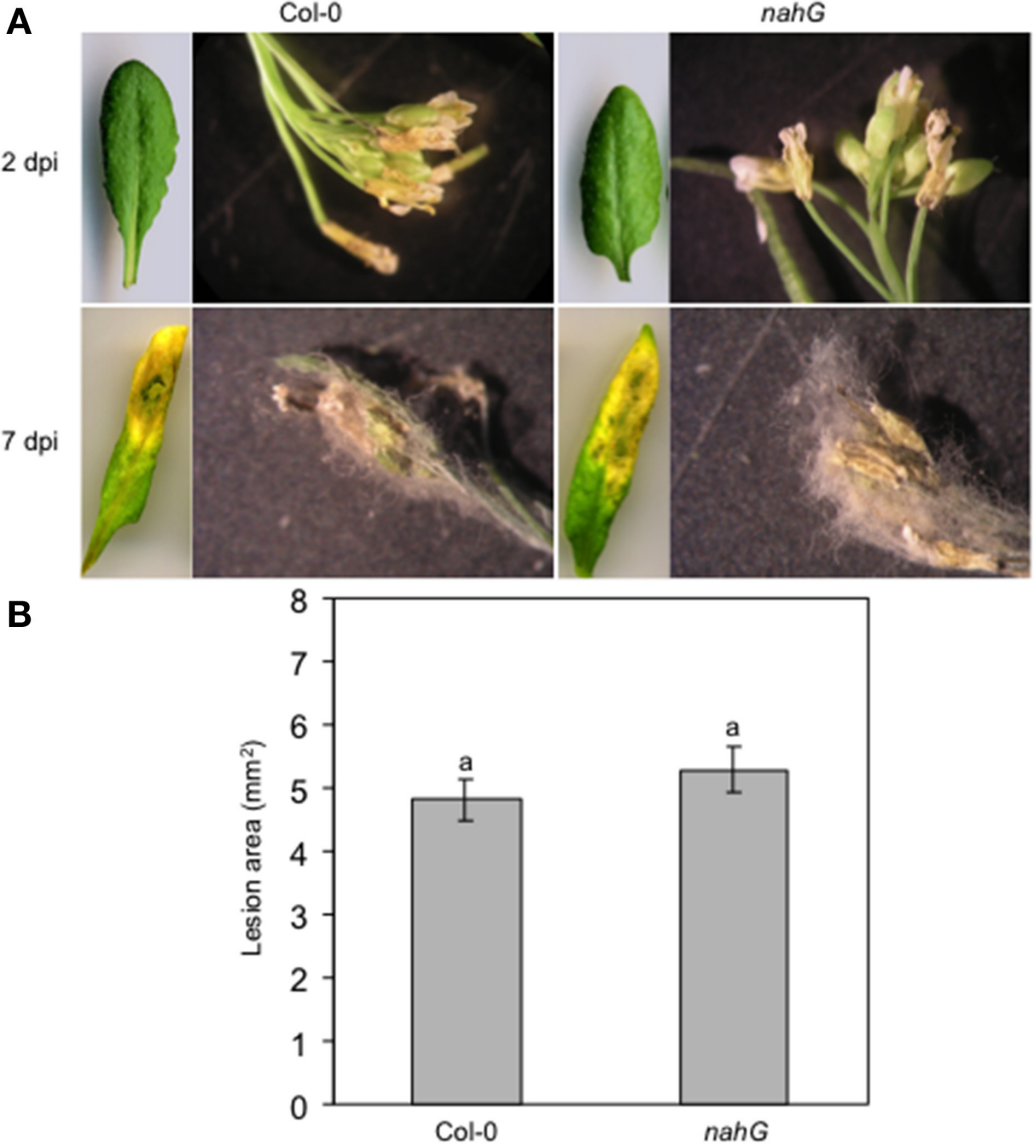
**Col-0 and n*ahG* response to the necrotrophic pathogen *Botrytis cinerea*. (A)** Progression over time of symptoms and signs on *B. cinerea* spray inoculated Col-0 and *nahG* leaves and flowers (see the legend of Figure 3 for sampling of plant material). On both genotypes, no leaf symptoms were detectable at 2 dpi while at 7 dpi chlorosis, necrosis and rot affected almost the entire leaf surface. At 2 dpi clear symptoms are seen in Col-0 and *nahG* flowers. At 7 dpi the pathogen completely covers the flower. The experiment was repeated 3 times with similar results. Ten replicates were used in each experiment. **(B)** Lesion area measured at 3 dpi in Col-0 and *nahG* leaves inoculated by placing a two 5 μL drop of conidial suspension on the upper surface of detached leaves. The experiment was repeated 3 times and resulted in 84 lesions observed on 42 leaves in each genotype. Bars represent the mean ± SE and different letters indicate statistically significant differences using Duncan's multiple range test (*P* ≤ 0.01).

To further investigate possible different responses of the floral organs and, in particular, of the sepals against necrotrophic pathogens, the Col-0 and SA-deficient *nahG* plants were spray-inoculated with *Botrytis cinerea* and the lesion development was measured. Visual assessment of spray-inoculated Arabidopsis plants at 2 dpi showed no symptoms on Col-0 and *nahG* leaves. In contrast, sepals and petals of both genotypes were necrotic. Moreover, gray mycelia started to appear on the floral surface (Figure 6A). At 7 dpi, the gray fungal structures completely covered the inflorescence of both genotypes. At the same time, clear symptoms (chlorosis and rot) were detectable on both Col-0 and *nahG* leaves (Figure 6A) and no difference in susceptibility was noted between Col-0 and *nahG* plants (Figure 6A). The lack of a significant difference in susceptibility of Col-0 and *nahG* leaves to *B. cinerea* was confirmed by lesion area measurements on drop-inoculated detached leaves at 3 dpi (Figure 6B). Thus, in this system, in contrast to the responses against the biotroph, the susceptibility to the necrotroph *B. cinerea* showed no differences between flowers and leaves, and between the two genotypes, which suggests a limited role for SA in the *B. cinerea* defense response.

## Discussion

The first indication of the presence of PR proteins in the flower comes from work on tobacco and, perhaps most importantly, it was demonstrated that there is a pathogen-independent induction (Lotan et al., 1989) and that PR proteins in the sepal are induced as part of the developmental program. More recently it was shown that tobacco and petunia contain chemically distinct floral defensins, basic small proteins that can retard the growth of fungi, oomycetes, and gram-positive bacteria. These defense mechanisms are specifically induced during the early stages of flower development (Lay et al., 2003) and operate in the outermost cell layers of sepals, petals, anthers, and styles, where they presumably serve in the first line of defense against pathogens.

Given these observations we hypothesized that the sepal may serve not just as a physical but also as a chemical defense barrier that protects the developing reproductive organs. The sepal has a very distinct morphology that includes polyploid giant cells (Figure 1). The biological role of these giant cells of the sepal is still not clear, but we know that they are the result of endoreduplication (Roeder et al., 2010). Endoploidy is essential for normal development and physiology in many different organisms. There are cells that go into endoreplication as part of terminal differentiation to enable specialized function (Lee et al., 2009). Plants grow by increasing cell numbers, cell size or both. Since increased DNA content correlates with increased cell size, endoreplication is a highly efficient growth mode since it reduces the cell (and cell wall) surface to volume ratio. Thus, such a growth mode may be particularly desirable when rapid growth must occur or high metabolic activity is required (Inzé and De Veylder, 2006). It is generally assumed that endoreplication-associated growth is indicative for cell types that perform specific biological functions. It has recently been reported that in Arabidopsis increasing gene copy number by localized endoreduplication, mediated by MYB3R4 (AT5G11510), may serve as a mechanism to meet the enhanced metabolic demands imposed by, e.g., the pathogenic biotroph fungus *Golovinomyces orontii* that depends entirely on the nutrient supply from the host (Chandran et al., 2010). Furthermore, there are numerous examples in other systems where specific cell types undergo endoreduplication and interestingly, it appears that these cell types often have roles in secretion and in some cases secretion of antimicrobial compounds (Reilly et al., 1994; Dai et al., 2010). For these reasons and based on the transcriptional profiles (Tables 1–6), we hypothesize that the sepal is in an heightened state of bio-chemical defense with many genes induced that encode enzymes that catalyze the production of defense components targeted for secretion into the extracellular space. In addition, we propose that the giant cells of the sepal are actually the metabolic factories that are synthesizing the defensive proteins as a preventive and/or protective measure. SA is a well-characterized molecule that regulates the activation and potentiation of plant defense responses (Vlot et al., 2009). However, the activation of defense responses is not the only regulatory role of SA. The first reported physiological responses to SA were the induction of thermogenesis through the activation of the mitochondrial alternative oxidase in Arum flowers (Raskin et al., 1987, 1989). Since then several other functions have been assigned to SA, including a regulatory role in flowering (Martinez et al., 2004), regulation of gene expression during leaf senescence (Morris et al., 2000) and regulation of cell growth by specifically affecting cell enlargement, endoreduplication and/or cell division. In addition, methyl SA is a volatile compound, and like other methyl esters (e.g., methyl benzoate, methyl cinnamate, methyl jasmonate) it is a widespread fragrant component in the plant kingdom contributing significantly to the floral scent output (Knudsen and Tollsten, 1993). Our data show very high constitutive levels of free and conjugated SA in both sepals and petals, whereas in pathogen-unchallenged leaves the SA content was very low. The high SA levels correlate well with the up-regulation of PRs protein encoding genes revealed by the transcriptome analysis.

We challenged leaves and inflorescence of Col-0 and *nahG* plants with the biotrophic fungus *G. cichoracearum* and the necrotrophic *B. cinerea* with the aim to clarify whether the defense program against these pathogens is enhanced in the inflorescence with respect to the leaves. When the leaves were infected with the fungus *G. cichoracearum*, the SA-deficient transgenic *nahG* plants were more sensitive than Col-0 and this is consistent with the SA-dependent resistance against the biotrophic pathogen in Arabidopsis leaves (Ellis et al., 2002). However, sepals and petals of Col-0 and *nahG* are both resistant to the biotroph with no or very few colonies forming on petals and sepals, respectively. Moreover, *G. cichoracearum* did not develop conidiphores and conidia on sepals. Since sepals and petals of Col-0 and SA-deficient plants were resistant to *G. cichoracearum*, we argue that in these organs the resistance to biotrophic pathogen is not exclusively or critically dependent on SA but depends on constitutive up-regulation of stress-responsive genes in flower. This interpretation is supported by the transcriptional analysis that shows a high induction of genes encoding proteins with a role in responses to chemical stimuli in the sepal (stage 12) and genes encoding proteins with a role in responses to biotic and chemical stimuli (stage 15). A comparison between the highly up-regulated genes in the sepal as compared to those in the petal reveals an enrichment in the categories “Catalytic activity” and “Response to stress” again point to an organ-specific and enhanced defense program (Table 3). *NahG* plants do not accumulate SA or camalexin (Nawrath and Metraux, 1999). However, there are many reports that show PRs activation after pathogen infection (Nawrath and Metraux, 1999; Govrin and Levine, 2002) thus suggesting that also in *nahG* mutant flowers there is a constitutive activation of defense mechanisms able to protect the flower against biotrophic pathogen infection. This point remains to be further elucidated and will be resolved when the transcriptome of *nahG* sepals becomes available.

Given the short lifespan of sepals and petals, and the possibility of senescence-related defense responses, we compared the sepal and petal transcriptomes to the transcriptome of senescent leaves (Tables 5, 6). In the case of the sepal, we noted enrichment in genes encoding proteins with catalytic activity (Tables 5 and Table S1) and in the case of the petal, an overrepresentation of genes encoding proteins with a role in cell wall function and external encapsulating structures (Tables 6 and Table S1). These results point to specific functions beyond the leaf senescence program and are conceivably indicators of enhanced functional and structural defense components. Additionally, the Arabidopsis petal with its absence of photosynthesis (Pyke and Page, 1998) is hardly particularly attractive to biotrophic pathogens.

In contrast to the response to the biotroph pathogen, the response to the necrotroph *B. cinerea* showed no difference between the flower and the leaves. In addition, the *B. cinerea* phenotype of *nahG* was indistinguishable from Col-0 suggesting a limited role for SA in the interaction between *B. cinerea* and Col-0 in Arabidopsis. Contrary to previous reports (Govrin and Levine, 2002; Ferrari et al., 2003), in our experiments *nahG* plants had resistance to *B. cinerea* that was comparable to Col-0 plants as previously reported (Thomma et al., 1998; Veronese et al., 2004; Abuqamar et al., 2006). These discrepancies may be caused by differences in the *Botrytis* strain or the methods of inoculation. In conclusion, the same *B. cinerea* disease susceptibility of leaves and flowers and Col-0 and *nahG* plants suggests that neither constitutive stress-responsive gene induction in sepals nor SA accumulation can prevent infections by this necrotrophic pathogen. On the other hand, induced defense responses to pathogens are mediated by multiple signal transduction pathways. While SA-mediated defenses are prominent against biotrophic pathogens, jasmonate/ethylene signaling exerts a major influence on plant response to necrotrophic pathogens such as *Botrytis cinerea* (Thomma et al., 1998, 1999). In addition, elicitors released from the cell wall during pathogen infection and genes involved in the biosynthesis of secondary metabolites play an important role in determining the enhanced resistance against *B. cinerea* through a signaling pathway activated by pathogen-associated molecular pattern molecules and, therefore, independently of SA, JA, and ET (Ferrari et al., 2007). Furthermore, it has been demonstrated that *B. cinerea* can induce multiple defense responses in Arabidopsis resembling to hypersensitive response (HR) (Govrin and Levine, 2002), but contrary to the effect on biotrophic pathogens, HR facilitates rapid growth and spread of this necrotrophyic pathogen.

In summary, we report that the sepal and petal express a distinct set of genes that encode proteins with a role in defense against pathogens. This tissue specific transcriptional program is reflected in the enhanced host responses, in particular to biotrophic pathogens. We also propose that the giant cells in the sepal are the metabolic factories that provide the chemical defense shield and we are currently planning to experimentally test this hypothesis.

## Author contributions

CG, SP, LE, MQ, and LX conceived and designed the experiments. LE and MQ performed the experiments. AD and CG performed the bioinformatics analyses. LE, SP, AD, and CG analyzed the data. CG and SP wrote the paper.

### Conflict of interest statement

The authors declare that the research was conducted in the absence of any commercial or financial relationships that could be construed as a potential conflict of interest.

## Supplementary material

Table S1 **Sepal gene expression at different stages**.

## Abbreviations

SA: salicylic acid
*nahG*: salicylate hydroxylase
SAGT: SA glucosyltransferase
GO: gene ontology
RCS: response to chemical stimulus
CMP: cellular metabolic process
RBS: response to biotic stimulus
RCS: response to chemical stimulus
ROO: response to other organisms
LRRK: leucine-rich receptor kinase
GA: gibberellic acid
dpi: days post-inoculation.
