# Supplementary material for: Arabidopsis flower specific defense gene expression patterns affect resistance to pathogens
Source: Front Plant Sci. 2015 Feb 20;6:79. doi: 10.3389/fpls.2015.00079 (PMC4335275; doi:10.3389/fpls.2015.00079)
Supplement: Supplementary file 2 [file Image1.PDF]

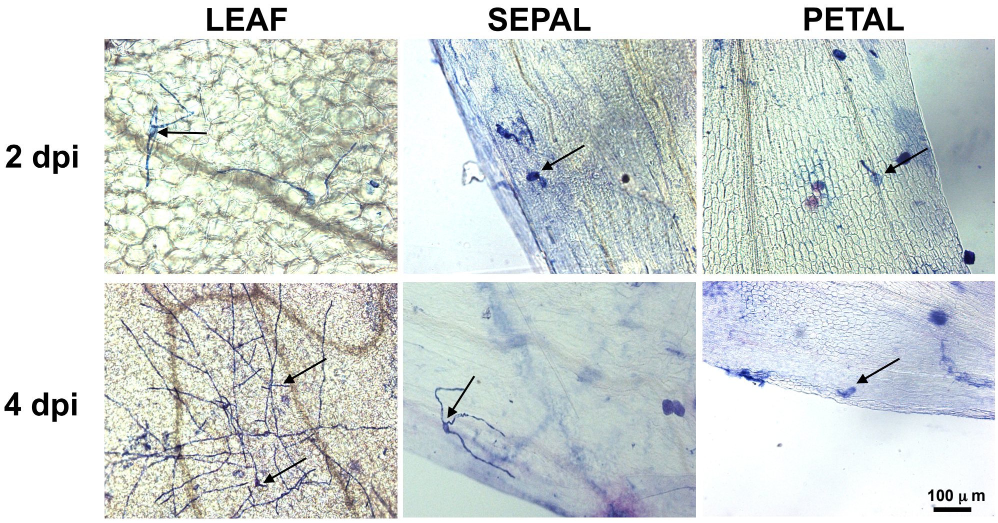

Trypan blue staining for the detection of *Golovinomyces cichoracearum* in Arabidopsis *nahG* mutant. Representative microscopic images of detached leaves, sepals and petals from *nahG* plants stained with Trypan blue at 2 and 4 days post spray inoculation with a conidial suspension of *G. cichoaraceraum*. Arrows indicate conidia that have just germinated and developed colonies on leaves at 2 and 4 dpi, respectively, and un-germinated or just germinated conidia on sepals and petals also at 2 and 4 dpi.
